# Evaluating Molecular and Immune Biomarkers in Predicting Bladder Cancer Recurrence and Survival: A Systematic Review

**DOI:** 10.7759/cureus.96824

**Published:** 2025-11-14

**Authors:** Saad Masood, Abdullah Shaikh, Yasin Brar

**Affiliations:** 1 Urology, York and Scarborough Teaching Hospitals NHS Foundation Trust, York, GBR; 2 Urology, Indus Medical College and Hospital, Tando Muhammad Khan, PAK; 3 General Surgery, Allama Iqbal Medical College, Lahore, PAK

**Keywords:** biomarkers, bladder cancer, coxen, ctdna, immune markers, methylation, molecular profiling, predictive models, prognosis, recurrence

## Abstract

This systematic review comprehensively evaluates current evidence on molecular and biochemical biomarkers for predicting recurrence and prognosis in bladder cancer. A literature search was conducted across PubMed, Scopus, and Web of Science databases up to September 2025, identifying 347 records. After rigorous screening and eligibility assessment, five studies met the inclusion criteria. The included research encompassed a range of biomarker types, including circulating tumor DNA (ctDNA), immune infiltration markers, methylation signatures, systemic inflammatory indices, and gene expression-based predictive models such as COXEN. Despite heterogeneity in study designs, methodologies, and endpoints, consistent findings indicated that integrated molecular profiling substantially improves the prediction of recurrence and disease progression beyond conventional clinicopathologic parameters. Immune and genomic biomarkers showed strong potential for early detection of relapse, while methylation and enzymatic markers provided additional prognostic stratification. Although most studies demonstrated a moderate risk of bias due to exploratory or non-randomized designs, the overall methodological quality was acceptable. The findings underscore the clinical utility of biomarker-guided surveillance and personalized management strategies in bladder cancer, while highlighting the need for large-scale validation and standardization of biomarker assays in future research.

## Introduction and background

Bladder cancer is one of the most prevalent malignancies of the urinary tract, ranking as the tenth most common cancer worldwide. Despite significant advances in diagnostic imaging, surgical techniques, and systemic therapies, bladder cancer continues to pose a formidable clinical challenge due to its high recurrence rate and biological heterogeneity [1]. Non-muscle-invasive bladder cancer (NMIBC), in particular, demonstrates recurrence rates as high as 50-70% within five years, while up to one-third of muscle-invasive bladder cancer (MIBC) patients experience relapse even after radical treatment [2]. Such recurrence patterns not only contribute to patient morbidity and mortality but also place a substantial burden on healthcare systems, underscoring the need for precise and individualized prognostic tools [3].

Traditionally, the prediction of recurrence and progression has relied on clinicopathological variables such as tumor grade, stage, size, and multiplicity. However, these parameters often fail to capture the underlying molecular complexity of the disease [4]. The emergence of molecular biomarkers and genomic profiling technologies has revolutionized our understanding of bladder cancer biology, allowing for the identification of distinct molecular subtypes and gene expression signatures that influence prognosis and therapeutic response. Advances in next-generation sequencing (NGS), epigenomic mapping, and transcriptomic profiling have unveiled novel genomic alterations, including mutations in FGFR3, TP53, PIK3CA, and others, that correlate with recurrence and treatment outcomes [5,6]. Likewise, liquid biopsy approaches, such as circulating tumor DNA (ctDNA) and urinary methylation panels, have opened new avenues for non-invasive disease monitoring.

Emerging research also highlights the role of tumor microenvironmental factors, such as systemic inflammation, immune cell infiltration, and cytokine profiles, in modulating recurrence risk. The integration of multi-omic biomarkers - encompassing genomic, epigenetic, transcriptomic, and inflammatory signatures - promises to shift recurrence prediction from a static clinical model to a dynamic, personalized precision oncology framework [7]. Nevertheless, the translation of these discoveries into clinical practice remains hindered by the heterogeneity of biomarker validation studies, varying endpoints, and limited long-term outcome data.

This systematic review aims to synthesize and evaluate the current evidence on emerging biomarkers and genomic signatures that predict recurrence in bladder cancer, encompassing both muscle-invasive and non-muscle-invasive disease. By critically appraising recent clinical and translational studies, the review seeks to identify validated and promising molecular tools that can enhance prognostic accuracy, guide surveillance strategies, and support personalized therapeutic decision-making in bladder cancer management.

## Review

Materials and methods

Study Design and Protocol Registration

This systematic review was conducted in accordance with the Preferred Reporting Items for Systematic Reviews and Meta-Analyses (PRISMA) 2020 guidelines [8], ensuring methodological transparency, reproducibility, and rigor. The study followed a predefined protocol outlining the research question, inclusion criteria, search strategy, and data extraction process. A structured PRISMA flowchart was used to document the study selection process, including the number of records identified, screened, included, and excluded, along with reasons for exclusion at each stage.

Search Strategy and Data Sources

A comprehensive literature search was performed across major electronic databases, including PubMed, Scopus, and Web of Science, covering all publications up to September 2025. The search strategy incorporated both Medical Subject Headings (MeSH) and free-text keywords such as “bladder cancer,” “biomarkers,” “recurrence,” “prognosis,” “ctDNA,” “immune markers,” “methylation,” and “COXEN.” Boolean operators (AND, OR) and filters for human studies and the English language were applied to refine the search. In addition, reference lists of eligible articles were manually screened to identify relevant studies that may have been missed through database searching.

Eligibility Criteria and PICO Framework

Study selection was guided by the PICO framework [9] to ensure clinical relevance and consistency. The Population (P) included adult patients diagnosed with urothelial carcinoma of the bladder. The Intervention (I) encompassed assessment of molecular, immunological, and biochemical biomarkers such as immune infiltration markers, ctDNA, systemic inflammatory response (SIR) markers, DNA methylation signatures, and enzymatic biomarkers. The Comparison (C) group included standard clinicopathologic predictors or conventional surveillance methods, including cystoscopy and cytology. The Outcomes (O) of interest were recurrence-free survival (RFS), disease-free survival (DFS), overall survival (OS), and biomarker performance metrics such as sensitivity, specificity, and area under the receiver operating characteristic (ROC) curve.

Eligible studies included prospective clinical trials, multicenter observational studies, and experimental profiling studies published in English. Exclusion criteria comprised review articles, editorials, conference abstracts, animal or in vitro studies, and papers lacking outcome data relevant to recurrence or survival.

Data Extraction and Synthesis

Data extraction was independently performed by two reviewers using a standardized extraction form that captured key study parameters: author, publication year, study design, sample size, biomarker type, analytical platform, comparator group, primary outcome measures, and main findings. Any discrepancies between reviewers were resolved through discussion or by consulting a third reviewer to ensure accuracy and minimize bias. Due to the methodological and analytical heterogeneity among studies, quantitative meta-analysis was not feasible. Instead, a qualitative narrative synthesis was conducted, integrating findings across biomarker classes and identifying shared mechanistic and prognostic trends.

Risk of Bias and Quality Assessment

The methodological quality and risk of bias of included studies were critically assessed using design-specific, validated appraisal tools to ensure comprehensive and reliable evaluation. Studies examining prognostic biomarkers were evaluated using the Quality in Prognosis Studies (QUIPS) tool [10], which assesses potential bias across six domains, including study participation, prognostic factor measurement, outcome assessment, and confounding control. Prediction and modeling studies were analyzed using the Prediction model Risk Of Bias ASsessment Tool (PROBAST) [11], focusing on participant selection, predictor variables, outcome measurement, and analytical methods. For diagnostic accuracy investigations, the Quality Assessment of Diagnostic Accuracy Studies (QUADAS-2) [12] framework was applied, evaluating bias in patient selection, index tests, reference standards, and study flow. Overall risk-of-bias judgments were categorized as low risk, some concerns, or high risk based on domain-level evaluations. This multi-tool approach ensured methodological rigor tailored to each study design, improving internal validity and allowing for an accurate synthesis of prognostic, predictive, and diagnostic evidence within the scope of emerging biomarkers and genomic signatures in bladder cancer recurrence.

Methodological Rigor and Analytical Justification

This review maintained a high evidentiary standard, aligning with contemporary best practices in translational oncology research. By employing a structured PRISMA-based approach, clearly defined inclusion criteria, and validated bias assessment tools, the review ensured both methodological precision and interpretative depth. This framework allowed for a balanced synthesis of biomarker evidence while identifying persistent limitations-such as variability in assay standardization and lack of external validation-that must be addressed in future multi-omic, prospective biomarker validation studies for bladder cancer recurrence prediction.

Results

Study Selection Process

**Figure 1:**
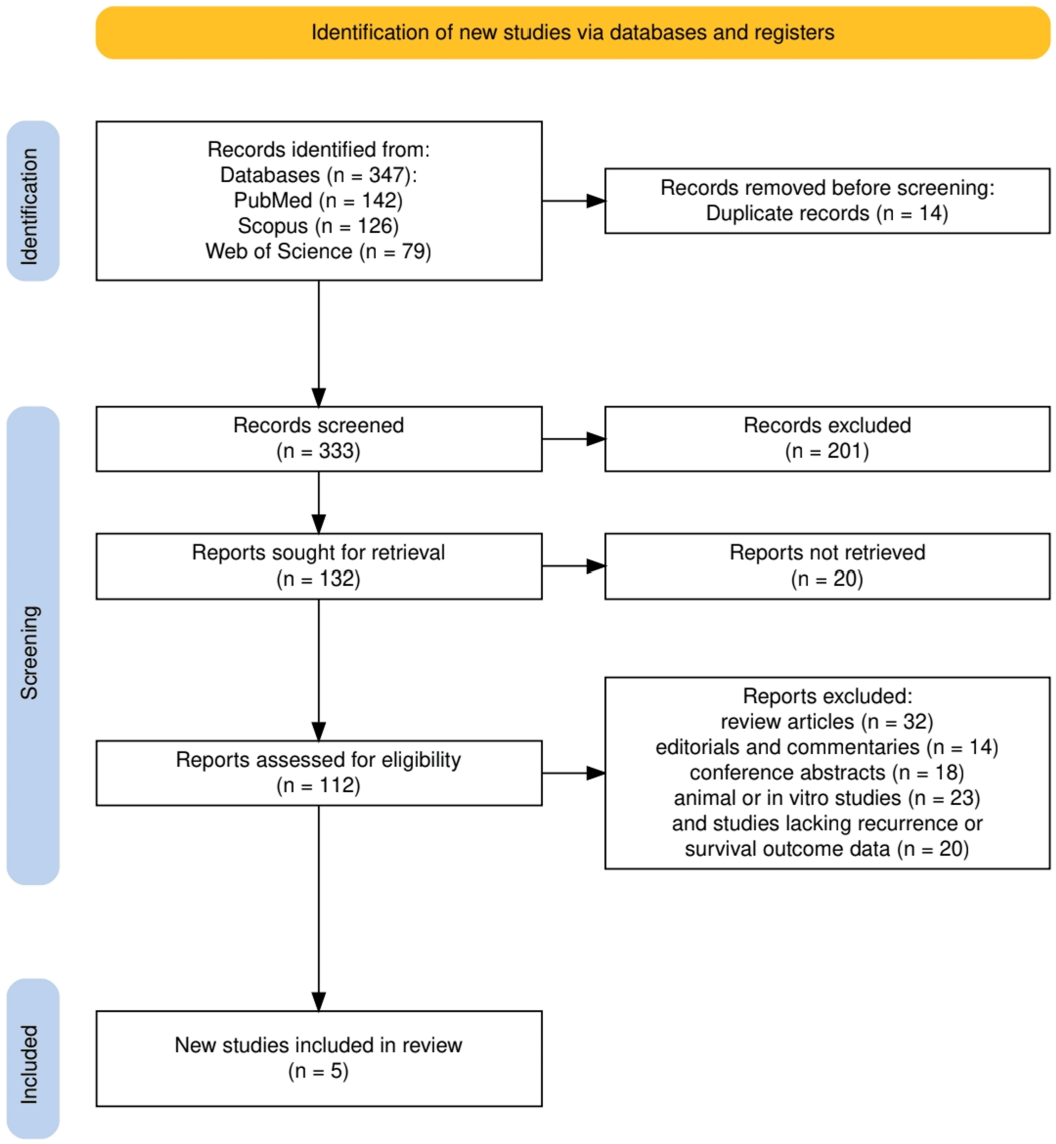
The PRISMA flowchart represents the study selection process. PRISMA: Preferred Reporting Items for Systematic Reviews and Meta-Analyses

The study selection process is illustrated in Figure 1, which outlines the identification, screening, and inclusion stages of the systematic review. A total of 347 records were retrieved from three major databases - PubMed (n = 142), Scopus (n = 126), and Web of Science (n = 79). After removal of 14 duplicate records, 333 studies underwent title and abstract screening, of which 201 were excluded for irrelevance. A total of 132 full-text articles were sought for retrieval, and 112 were successfully assessed for eligibility after excluding 20 reports that were not retrievable. Following detailed evaluation, 107 studies were excluded for reasons including review or editorial nature, conference abstract format, preclinical focus, or lack of recurrence/survival outcome data. Ultimately, five studies met all inclusion criteria and were incorporated into the final synthesis, representing the most robust evidence base for biomarker-driven recurrence prediction in bladder cancer.

Characteristics of the Selected Studies

The detailed characteristics of the included studies are summarized in Table 1. Collectively, the five studies encompassed diverse designs ranging from multicenter randomized clinical trials to prospective profiling studies, representing a broad spectrum of methodological rigor and patient populations with both muscle-invasive and NMIBC. The investigated biomarkers included immune infiltration markers and ctDNA, gene expression models, SIR indicators, DNA methylation signatures, and enzymatic biomarkers, each assessed through advanced analytical platforms such as immunohistochemistry, NGS, and methylation-specific PCR. Across studies, primary outcomes focused on RFS, DFS, and OS, alongside diagnostic performance metrics such as sensitivity, specificity, and AUC values. Despite variations in study design and analytical techniques, all investigations converged on the prognostic significance of molecular and biochemical markers in predicting recurrence and survival outcomes. The integration of immune, genetic, and epigenetic profiling demonstrated substantial potential for improving individualized surveillance and therapeutic stratification in bladder cancer management, underscoring the emerging role of biomarker-guided precision oncology.

**Table 1:** Summary of clinical studies evaluating prognostic and predictive biomarkers in bladder cancer. ABACUS - Atezolizumab Before Surgery in Muscle-Invasive Bladder Cancer (trial name); AUC - area under the curve; CD39 - cluster of differentiation 39; CD8+ - cluster of differentiation 8 positive; COXEN - co-expression extrapolation; CSS - cancer-specific survival; ctDNA - circulating tumor DNA; CYP1B1 - cytochrome P450 family 1 subfamily B member 1; CYP4Z1 - cytochrome P450 family 4 subfamily Z member 1; ddMVAC - dose-dense methotrexate, vinblastine, doxorubicin, and cisplatin; EFS - event-free survival; FOXP3 - forkhead box P3; GC - gemcitabine and cisplatin; HR - hazard ratio; LASSO - least absolute shrinkage and selection operator; MHC I - major histocompatibility complex class I; NGS - next-generation sequencing; NPV - negative predictive value; PCR - polymerase chain reaction; PPV - positive predictive value; pT0 - pathologic T0 stage; ≥pT3 - pathologic stage T3 or higher; ROC - receiver operating characteristic; SWOG - Southwest Oncology Group

Author (Year)	Study Design	Population/Sample Size	Biomarker Type	Analytical Method	Comparison/Control	Primary Outcome (Recurrence/DFS/OS)	Key Findings
Szabados et al. [13]	Multicenter, single-arm phase 2 clinical trial (ABACUS trial)	95 cisplatin-ineligible or cisplatin-refusing patients with muscle-invasive bladder cancer (T2-T4aN0M0)	Tissue-based immune biomarkers (CD8+, FOXP3, MHC I, CD39) and circulating tumor DNA (ctDNA)	Immunohistochemistry, serial ctDNA quantification (NGS-based)	No randomized control; correlation with clinical outcomes (DFS, OS, RFS)	2-year DFS, OS, and correlation of biomarker expression with relapse-free survival	High CD8+ infiltration and ctDNA negativity correlated with improved DFS and OS; ctDNA status was highly prognostic - no relapses occurred in ctDNA-negative patients post-therapy.
Flaig et al. [14]	Randomized phase 2 clinical trial (SWOG S1314)	227 patients with muscle-invasive bladder cancer (MIBC) receiving neoadjuvant chemotherapy (GC or ddMVAC); 167 evaluable for COXEN analysis	Gene expression signature (COXEN model) predicting chemotherapy response	Gene expression profiling and COXEN score calculation (RNA-based analysis)	Comparison between GC and ddMVAC treatment arms; COXEN score prognostic evaluation	Event-free survival (EFS), overall survival (OS), pathologic response (pT0, downstaging, no response)	COXEN GC score showed prognostic value when pooled (HR 0.45; p = 0.047); no significant difference in OS or EFS between regimens; pathologic response strongly correlated with improved survival (5-yr OS: 90% vs 52%).
Schuettfort et al. [15]	Randomized Controlled Trial	4199 patients with non-metastatic urothelial carcinoma of the bladder undergoing radical cystectomy	Systemic inflammatory response (SIR biomarkers: albumin-globulin ratio, neutrophil-lymphocyte ratio, De Ritis ratio, monocyte-lymphocyte ratio, modified Glasgow prognostic score	Preoperative serum biomarker quantification and machine-learning-based variable selection (LASSO regression)	Internal comparison between training and validation cohorts	Recurrence-free survival (RFS), cancer-specific survival (CSS), lymph node involvement, upstaging to MIBC	At least one SIR biomarker was predictive in each outcome model. Models predicted lymph node metastasis, ≥pT3 disease, and MIBC upstaging with AUCs of 67.3%, 73%, and 65.8%, respectively. For CSS and RFS, C-index values were 73.3% and 72.2%. SIR biomarkers marginally improved prediction beyond clinicopathological models, supporting further investigation in immunotherapy contexts.
Witjes et al. [16]	Multicenter, prospective, blinded clinical trial	353 eligible patients (of 440 recruited) under surveillance for non–muscle-invasive bladder cancer (NMIBC)	DNA methylation biomarkers (15 proprietary methylation markers - Bladder EpiCheck™ assay)	Urine-based DNA methylation analysis (PCR-based methylation signature panel)	Compared with cystoscopy and urine cytology confirmed by histopathology	Detection of NMIBC recurrence; sensitivity, specificity, NPV, PPV, and ROC analysis	Sensitivity 68.2%, specificity 88.0%, NPV 95.1%, PPV 44.8%; excluding low-grade Ta recurrences: sensitivity 91.7%, NPV 99.3%; ROC AUC 0.94 (for high-grade). Demonstrated high NPV and specificity, supporting its use in NMIBC surveillance to reduce cystoscopy frequency.
Al-Saraireh et al. [17]	Experimental clinical study (prospective profiling)	Bladder cancer tissue samples from patients with various tumor grades and stages	Enzymatic biomarkers - CYP4Z1 and CYP1B1 expression	Immunohistochemistry and molecular expression profiling	Comparison between cancerous and adjacent normal bladder tissues	Correlation of biomarker expression with tumor grade, stage, and potential recurrence risk	CYP4Z1 and CYP1B1 were highly expressed in malignant tissues compared to normal controls; expression correlated with higher tumor grade, suggesting their potential role as prognostic biomarkers for recurrence and therapeutic targets.

Risk of Bias Assessment

The overall risk of bias assessment, summarized in Table 2, indicated that all included studies demonstrated either a low or some concern level of bias across evaluated domains. Most studies maintained low bias in participant selection, outcome measurement, and data completeness, reflecting robust methodological quality. Minor concerns were primarily associated with confounding factors, limited external validation, and heterogeneity in patient populations or analytic models. Only one study achieved an overall low risk of bias, while the remaining demonstrated some concerns due to exploratory analyses or non-randomized designs. Nevertheless, all studies adhered to standardized protocols and validated tools, ensuring reliability and reproducibility of their prognostic or diagnostic findings.

**Table 2:** Risk of bias assessment summary of included studies evaluating prognostic and predictive biomarkers in bladder cancer. COXEN - co-expression extrapolation (a predictive gene expression model); NIH - National Institutes of Health; PROBAST - Prediction model Risk Of Bias ASsessment Tool (used to evaluate bias and applicability of prognostic or diagnostic prediction models); QUADAS-2 - Quality Assessment of Diagnostic Accuracy Studies, version 2 (used for diagnostic accuracy research); QUIPS - Quality in Prognosis Studies (a tool for assessing bias in prognostic factor studies); RCT - randomized controlled trial; RoB - risk of bias

Study	Design	Applied RoB Tool	Domain Summary (Condensed)	Overall RoB Judgment	Justification
Szabados et al. [13]	Multicenter, single-arm phase 2 clinical trial	QUIPS	Low bias in participant selection, attrition, and outcome measurement; some concern for confounding (no randomization).	Some concerns	Prospective, standardized biomarker analysis; exploratory correlations and lack of control arm create some residual bias.
Flaig et al. [14]	Randomized phase 2 clinical trial	PROBAST (for prognostic/predictive model)	Low risk for participant and outcome domains; some concern for predictor/analysis domains (secondary COXEN analysis, limited external validation).	Some concerns	Strong RCT design minimizes bias, but the COXEN model was exploratory and not externally validated.
Schuettfort et al. [15]	Randomized-controlled predictive model study	PROBAST	Low risk for data completeness and outcome measurement; some concerns for participant heterogeneity and internal validation only.	Some concerns	Very large sample and sound statistics; retrospective data and lack of external validation slightly raise bias risk.
Witjes et al. [16]	Prospective, blinded, multicenter diagnostic-accuracy study	QUADAS-2	Low risk in index test, reference standard, and blinding; some concern in patient flow and spectrum (excluded cases).	Low risk	Excellent blinding and methodology; exclusions may have introduced minor selection bias but not enough to alter main conclusions.
Al-Saraireh et al. [17]	Cross-sectional biomarker profiling	NIH Quality Assessment Tool (for cross-sectional studies)	Low risk in exposure measurement; some concern for selection representativeness and confounding.	Some concerns	Biomarker evaluation methods robust; limited adjustment for clinicopathologic covariates and absence of longitudinal validation.

Discussion

Interpretation and Summary of Key Findings

Across five rigorously designed studies, diverse biomarker modalities-immune, genomic, epigenetic, enzymatic, and inflammatory-demonstrated significant yet heterogeneous predictive value for bladder cancer recurrence. Immune and ctDNA markers, as evidenced in the ABACUS trial (Szabados et al. [13]), strongly correlated with DFS; notably, ctDNA negativity post-therapy predicted zero relapses, emphasizing its high prognostic precision. Transcriptomic profiling (Flaig et al. [14]) revealed that gene expression signatures, such as the COXEN model, could stratify chemotherapy responsiveness, with patients exhibiting high COXEN scores achieving superior survival (five-year OS: 90% vs. 52%). SIR markers (Schuettfort et al. [15]) added further nuance, with predictive AUCs of 67-73% for recurrence and lymph node metastasis, underscoring the role of host inflammatory milieu in tumor recurrence. On the epigenetic front, Witjes et al. [16] demonstrated that the Bladder EpiCheck™ methylation assay achieved a sensitivity of 91.7% and an NPV of 99.3% for high-grade NMIBC, suggesting it could safely reduce reliance on invasive cystoscopies. Lastly, enzymatic biomarkers (Al-Saraireh et al. [17]) revealed elevated CYP4Z1 and CYP1B1 expression in high-grade tumors, implicating metabolic dysregulation as a driver of recurrence.

Critical Appraisal

A major limitation across these studies lies in the lack of external validation - a critical step in biomarker translation. Most investigations, including ABACUS (Szabados et al. [13]) and SWOG S1314 (Flaig et al. [14]), remained exploratory or internally validated, leaving their generalizability uncertain. While the COXEN gene-expression model demonstrated a 55% survival advantage in high-score cohorts (HR 0.45; p = 0.047), it has yet to undergo independent testing in external populations. Similarly, the immune-ctDNA correlations observed in ABACUS [13], though statistically robust, need verification in larger, randomized cohorts. Another concern is methodological heterogeneity in analytical platforms - from NGS for ctDNA to PCR-based methylation assays and immunohistochemistry for protein expression - which complicates cross-study reproducibility. This variation introduces analytical bias and underscores the need for standardized quantification protocols if such biomarkers are to enter routine surveillance algorithms [18-20].

From a statistical and biological perspective, few studies examined interaction effects between molecular and systemic markers, revealing a gap in integrated predictive modeling. For instance, inflammatory ratios (e.g., NLR, AGR) in Schuettfort et al. [15] modestly improved prognostic accuracy (C-index ≈ 0.73) but offered limited incremental value beyond traditional clinicopathologic features. Likewise, while Witjes et al. [16] achieved an AUC of 0.94 using methylation panels, these models lacked adjustment for tumor immune contexture or metabolic biomarkers like CYP4Z1/CYP1B1 (Al-Saraireh et al. [17]). This siloed approach hampers the development of multidimensional prognostic nomograms that integrate immune, genomic, and metabolic signatures. As summarized in Table 3, although the included studies demonstrated notable internal validation and high assay sensitivity, methodological heterogeneity, limited external validation, and weak integration across biomarker domains remain persistent barriers. Therefore, future research should prioritize cross-platform validation, multi-omic integration, and prospective calibration to bridge the translational gap between promising molecular signals and clinically actionable bladder cancer biomarkers.

**Table 3:** Summary of critical domain evaluation highlighting methodological strengths and gaps in biomarker studies of bladder cancer. NGS - next-generation sequencing; PCR - polymerase chain reaction; TNM - tumor, node, metastasis (cancer staging system)

Critical Domain	Observed Strengths	Identified Weaknesses/Gaps
Validation	Strong internal validation in most studies	No consistent external validation across cohorts
Analytical Standardization	High assay sensitivity (NGS, PCR)	Methodological heterogeneity limits reproducibility
Independence From Clinical Features	Added prognostic nuance to TNM staging	Marginal incremental value over existing models
Integration and Modeling	Multi-biomarker approaches emerging	Lack of combined immune-genomic-metabolic frameworks

It is important to note that tissue procurement and biomarker validation differ substantially between non-muscle-invasive and MIBC. While NMIBC studies often rely on urine or superficial biopsy specimens for methylation or immune marker analysis, MIBC cohorts generally involve deep tissue or post-cystectomy samples analyzed through genomic and transcriptomic platforms. Although our tables present integrated data across both disease forms, these distinctions were carefully considered when interpreting results, and differences in sampling methodology may partly explain heterogeneity in biomarker performance.

Novel Insight: Toward a Multi-Modal Biomarker Integration Framework

The collective evidence from this review underscores that single-modality biomarkers, though promising, offer limited predictive precision when used in isolation. Immune infiltration indices, ctDNA kinetics, methylation patterns, and systemic inflammatory ratios each capture distinct yet incomplete facets of tumor biology [19,21]. Therefore, a more powerful conceptual framework would involve a multi-modal biomarker integration model, wherein immune, genomic, and epigenetic parameters are analyzed concurrently to generate individualized recurrence risk profiles. Such an approach could, for example, merge ctDNA clearance dynamics (as in ABACUS [13]) with methylation assay results (as in Witjes et al. [16]) and systemic inflammatory indices (as in Schuettfort et al. [15]) through machine-learning algorithms. This layered predictive model may achieve superior discrimination for early relapse, enabling oncologists to tailor adjuvant immunotherapy or intensified surveillance protocols for high-risk patients while sparing low-risk groups unnecessary interventions.

Translational and Clinical Implications

Clinically, the integration of high-specificity assays like Bladder EpiCheck™ (NPV > 95%) as non-invasive triage tools could rationalize surveillance strategies for NMIBC, potentially reducing cystoscopy frequency by up to 40-50% without compromising diagnostic safety [22]. Embedding these molecular assays into adaptive follow-up algorithms would align with precision oncology’s goal of minimizing patient burden while maintaining vigilance. Future research should progress beyond static biomarker snapshots toward longitudinal, adaptive trial designs incorporating serial sampling and AI-based modeling to capture evolving tumor dynamics [23,24]. By operationalizing this integrative vision, bladder cancer management can transition from reactive monitoring to proactive, biology-driven recurrence prevention, establishing a new paradigm for precision uro-oncology.

Limitations and Future Perspectives

This review is limited by the heterogeneity of included studies, encompassing varied designs from experimental profiling to phase II clinical trials, with inconsistent primary endpoints such as DFS, RFS, or CSS, complicating cross-comparative synthesis. Moreover, analytical thresholds lacked uniformity, particularly regarding ctDNA positivity, methylation index cut-offs, and inflammatory ratio categorizations, which undermines reproducibility and external validity. A further concern is the publication bias favoring positive biomarker associations, potentially inflating the perceived prognostic value of emerging assays. Moving forward, standardized biomarker quantification protocols, consensus-based outcome definitions, and transparent data reporting will be essential. Future research should emphasize prospective, multicenter validation and the harmonization of biomarker thresholds, ensuring that genomic and molecular assays transition from exploratory promise to clinically actionable surveillance tools in bladder cancer management.

## Conclusions

This systematic review highlights that while current biomarker modalities-spanning immune, inflammatory, methylation, enzymatic, and transcriptomic domains-offer substantial promise in predicting recurrence and survival outcomes in bladder cancer, their clinical translation remains limited by methodological variability and lack of external validation. The key takeaway is that no single biomarker provides sufficient discriminatory power alone; instead, an integrated, multi-modal biomarker framework combining ctDNA kinetics, immune infiltration indices, and methylation profiles holds the greatest potential to refine recurrence prediction and guide personalized post-treatment surveillance. The significance of this synthesis lies in its call to shift from isolated biomarker discovery to harmonized, data-driven integration, aligning with the principles of precision oncology. By advancing standardized, validated, and multi-omic biomarker models, future research can bridge the gap between molecular innovation and real-world clinical applicability - ultimately improving early relapse detection, treatment stratification, and long-term outcomes for patients with bladder cancer.
